# Changes in hospitalized coronavirus disease 2019 (COVID-19) patient characteristics and resource use in a system of community hospitals in the United States

**DOI:** 10.1017/ice.2020.1264

**Published:** 2020-10-12

**Authors:** Kenneth E. Sands, Richard P. Wenzel, Laura E. McLean, Kimberly M. Korwek, Jonathon D. Roach, Russell E. Poland, Karla M. Miller, L. Hayley Burgess, Edmund S. Jackson, Jonathan B. Perlin

**Affiliations:** 1 Clinical Services Group, HCA Healthcare, Nashville, Tennessee; 2 Department of Internal Medicine, Virginia Commonwealth University Medical Center, Richmond, Virginia

## Abstract

Coronavirus disease 2019 (COVID-19) has migrated to regions that were initially spared, and it is likely that different populations are currently at risk for illness. Herein, we present our observations of the change in characteristics and resource use of COVID-19 patients over time in a national system of community hospitals to help inform those managing surge planning, operational management, and future policy decisions.

## Methods

Demographic data were collected from the electronic health records of inpatients with discharged from facilities affiliated with a large healthcare system. The HCA Healthcare system consists of 186 hospitals and >2,000 sites of care located in 21 states and the United Kingdom. Acute-care facilities consist primarily of urban and suburban community hospitals as well as specialty and tertiary referral facilities. Collectively, these facilities provide ~5% of all inpatient hospital services in the United States. Affiliated facilities are distributed geographically across the country with a concentration in the southern United States.

For this analysis, patients were included if they had laboratory-confirmed COVID-19 (using reverse-transcriptase polymerase chain reaction (RT-PCR) for severe acute respiratory coronavirus virus 2 (SARS-CoV-2)] and were discharged between March 1 and June 30, 2020 (N = 14,165). Aggregated data for age, race, ethnicity, length of stay, level of care, discharge disposition, and select treatments were analyzed by month.

## Results

Monthly volumes of hospitalized SARS-CoV-2–positive patients increased steadily between March and June, 2020 (Table 1) while the average age decreased (March to June, −11.96 years, *P* < .0001). The distribution of patient age also changed from a unimodal, nearly normal distribution in March with a peak between 44 and 64 years of age, to a bimodal and right-skewed distribution in June with peaks around 24–34 years and 44–54 years.

**Table 1.** Characteristics of Discharged COVID-19 Patients by Month

Discharge Month	Total Discharged COVID-19 Patients	Mean Patient Age, Years	Mean LOS, Days	% Male	% Hispanic Ethnicity	Overall Mortality Rate, %	% of COVID-19 Patients Receiving Selected Treatment			
							Hydroxy-chloroquine	Tocilizumab	Remdesivir	Steroids
March	769	58.1	4.4	51.0	19.0	15.6	37.3	0.4	1.1	15.9
April	3,840	62.0	7.3	52.9	25.5	23.1	57.9	3.7	0.6	25.4
May	3,637	61.1	8.9	50.5	33.2	15.5	27.8	8.2	5.0	31.3
June	5,919	56.3	6.7	50.8	41.0	9.9	5.4	5.2	21.3	34.0

The distribution of patient race and ethnicity also varied over time, which may reflect the change in distribution of active COVID-19 surges and the service areas of affiliated facilities. Most notably, there was a substantial increase in the number of patients who identified as Hispanic ethnicity (Table 1). This coincides with the increase in viral activity in Texas and Florida during June, and the large representation of this healthcare system in those areas.

Among hospitalized COVID-19 patients, the mean length of stay (LOS) generally increases with age. This trend held true over the 4-month time period, even as the mean LOS changed by month (Table 1). The median LOS for patients who identified as Hispanic ethnicity was ~1 day shorter than that for patients who did not identify as Hispanic; however, Hispanic patients were on average younger (mean, 52 years).

In March, patients initially admitted to non–critical care units who did not require critical care or mechanical ventilation during their encounter accounted for 622 of 769 of hospitalized COVID-19 patients (80.9%). By June, patients meeting this criteria accounted for 4,671 of 5,919 patients (78.9%).

When segregated by month, patient age bracket (>65 years or <65 years), and severity at admission, the mortality rate declined month after month for all groups. For patients <65 years of age who were initially admitted to a noncritical care unit, the mortality rate decreased from 5.0% (83 of 1,657) in April to 2.1% (64 of 3,063) in June. For patients >65 years of age initially admitted to a noncritical care unit, the mortality rate decreased from 29.4% (339 of 1,358) in April to 13.8% (222 of 1,608) in June.

Between March and June, the use of hydroxychloroquine decreased precipitously, consistent with the revised recommendations against this therapy (Table 1).^1^ The use of tocilizumab remained relatively constant and rare, consistent with the use of this treatment in patients with severe disease. The use of remdesivir increased, but this may be attenuated by access issues (eg, clinical trial participation, compassionate use, or government allocation required). Since March, ~40% of all COVID-19 patients received treatment with tocilizumab, remdesivir, or steroids (alone or in combination).

## Discussion

As the COVID-19 pandemic continues, there are observable changes in the patient population affected, hospitalization and resource use, and mortality rate. Using data from COVID-19 patients admitted to community hospitals across the United States during nonsurge conditions, we showed that although patient age and other characteristics changed over time, outcomes are improving for all patients. This finding suggests that knowledge gained from early experiences with this disease is allowing providers to better care for patients with COVID-19.
